# Knockdown of TRIM52 alleviates LPS-induced inflammatory injury in human periodontal ligament cells through the TLR4/NF-κB pathway

**DOI:** 10.1042/BSR20201223

**Published:** 2020-08-10

**Authors:** Peng Liu, Lijun Cui, Lifang Shen

**Affiliations:** 1 Department of Prosthodontics, Shanxi Provincial People’s Hospital, Taiyuan, Shanxi Province, China; 2 Department of Stomatology, Cui Lijun Dental Clinic, Datong, Shanxi Province, China; 3 Department of Stomatology, Meiyuan Stomatological Hospital, Datong, Shanxi Province, China

**Keywords:** nuclear factor-kappa B, periodontitis, toll like receptor 4, tripartite motif containing 52

## Abstract

Tripartite motif-containing (TRIM) 52 (TRIM52) is a vital regulator of inflammation. However, the function and mechanisms of TRIM52 in lipopolysaccharide (LPS)-induced inflammatory injury of human periodontal ligament cells (HPDLCs) in periodontitis remain undefined. In the present research, gene expression was determined using a quantitative polymerase chain reaction and Western blot. The effect of TRIM52 on LPS-induced inflammatory injury was evaluated using 3-(4,5-dimethylthiazol-2-yl)-2,5-diphenyltetrazolium bromide (MTT) assay, flow cytometry, and enyzme-linked immunosorbent assay (ELISA). We found that TRIM52 expression was up-regulated in LPS-treated HPDLCs. Knockdown of TRIM52 alleviated LPS-induced proliferative inhibition and apoptosis promotion in HPDLCs, as evidenced by a decrease in cleaved caspase-3 expression and caspase-3 activity. Silencing TRIM52 suppressed LPS-induced inflammatory response of HPDLCs, as indicated by the decrease in interleukin (IL)-6, IL-8, tumor necrosis factor-α (TNF-α) levels, and increase in IL-10 levels. TRIM52 knockdown inhibited LPS-induced activation of TLR4/nuclear factor-κ B (NF-κB) signaling pathway. Taken together, knockdown of TRIM52 mitigated LPS-induced inflammatory injury via the TLR4/NF-κB signaling pathway, providing an effective therapeutic target for periodontitis.

## Introduction

Periodontitis is a chronic infective disease of periodontal tissues, with the characteristics of periodontal destruction, periodontal pockets formation, progressive attachment loss and frontal resorption [1]. Periodontitis is mainly triggered by bacteria in dental plaque, which is the major reason for tooth loss [2]. Nowadays, periodontitis has become the second most common oral disease, and approximately 50% of the population suffers from this disease throughout the world [3,4]. However, the pathogenesis of periodontitis has not yet been elucidated. As such, clarification of the mechanism of periodontitis is of importance to develop a novel and effective therapy for periodontitis.

Periodontitis is a chronic inflammatory disease, which is affected by multiple factors [5]. Recently, dental plaque was recognized as the major factor leading to periodontitis. Lipopolysaccharide (LPS), also called endotoxin, is one of the important components of Gram-negative bacteria cell wall. LPS is also a potent activator of inflammatory responses, leading to the overproduction of inflammatory factors, such as interleukin (IL)-6, IL-8 and tumor necrosis factor-α (TNF-α) [6]. Reportedly, the overexpression of inflammatory factor is a key determinant of alveolar bone resorption and periodontal attachment loss. Therefore, LPS was widely used to induce the cell model of periodontitis and inhibition of LPS-induced inflammatory injury is key to the treatment of periodontitis.

The nuclear factor-κB (NF-κB) can be activated in response to various intracellular or extracellular stimuli, and its disorder can lead to pathological conditions such as infection, inflammation and immune disorders [7]. Studies have shown that inhibiting the activation of the NF-κB pathway helps promote periodontal tissue repair in experimental periodontitis rats [8]. Tripartite motif-containing (TRIM) is an evolutionarily ancient protein family that plays a principal factor in a wide range of cellular processes, such as tumorigenesis and virus or bacteria immune defense [9–12]. As a member of the RING-type ubiquitin E3 ligase, the role of TRIM family proteins in regulating the NF-κB pathway has been extensively studied [13]. As an example, *Mycobacterium tuberculosis* (Mtb) infection induced up-regulation of TRIM22, while knockdown of TRIM22 inhibited the autophagy and promoted bacterial survival of Mtb-infected THP-1 cells by regulating the NF-κB/beclin 1 signaling [14]. Among TRIM family members, TRIM52 has been identified as a novel antiviral gene [15]. Also, TRIM52 was down-regulated in hepatocellular carcinoma tissues and cell lines (MHCC-97H and MHCC-97L), and its silencing could repress cell proliferation, migration and invasion, while induce cell cycle arrest in MHCC-97H cells through inhibiting the ubiquitination of protein phosphatase Mg/Mn-dependent 1A [16]. However, the role of TRIM52 in regulating LPS-induced NF-κB activation has not been explored. Moreover, little is known about the role of TRIM52 in the development of periodontitis. The present study further enriched our knowledge of the mechanism in the pathogenesis of periodontitis.

In the present study, we aimed to investigate the function and mechanism of TRIM52 in LPS-induced inflammatory injury in human periodontal ligament cells (HPDLCs). Our findings suggested that LPS treatment induced the up-regulation of TRIM52 in HPDLCs. Mechanically, silencing of TRIM52 mitigated LPS-induced proliferative inhibition, apoptosis promotion and inflammatory response in HPDLCs via TLR4/NF-κB pathway. Targeting TRIM52 may become an attractive strategy for the treatment of inflammatory diseases, including periodontitis.

## Materials and methods

### Cell culture and treatment

HPDLCs were incubated in Dulbecco’s modified Eagle’s medium (DMEM) medium plus 10% fetal bovine serum (FBS) and penicillin–streptomycin liquid in a carbon dioxide incubator with 5% CO_2_ at 37°C. HPDLCs were detached with trypsin when they reached confluence. HPDLCs were treated with PBS or LPS (0.5 or 1 μg/ml). After time intervals of 12 or 24 h, HPDLCs were collected for the following experiments.

The small interfering RNA (siRNA) sequences targeting TRIM52 (si-TRIM52-1 and si-TRIM52-2) and sequence-scrambled siRNA (si-NC) were synthesized by RiboBio (Guangzhou, China). All transfection reactions were performed using Lipofectamine 2000 (Invitrogen, Carlsbad, CA, U.S.A.), following the manufacturer’s specifications.

### Quantitative real-time polymerase chain reaction analysis

Total RNA was isolated from HPDLCs with help of TRIzol reagent from Invitrogen followed by reverse transcription into cDNA using a High Capacity cDNA Reverse Transcription Kit from Applied Biosystems (Carlsbad, CA, U.S.A.). qPCR analysis was performed on an ABIPrism 7900HT Real-Time System (Applied Biosystems, Carlsbad, CA, U.S.A.) using the SYBR Green qPCR Master Mix from Applied Biosystems. The expression of TRIM52, TLR4, NF-κB p65, IL-6, IL-8, TNF-α and IL-10 were analyzed using the 2^−ΔΔ*C*^_t_ method with the β-actin as an internal reference. Primer sequences for these genes were listed as follows: TRIM52 forward: 5′-GCCATCTGCTTGGATTACTTC-3′, and reverse: 5′-TTCATCTTCCTCCTCGTTCTG-3′; TLR4 forward: 5′-CACAGACTTGCGGGTTCTACATC-3′, and reverse: 5′-AGTTCATAGGGTTCAGGGACAGG-3′; NF-κB p65 forward: 5′-AGGCAAGGAATAATGCTGTCCTG-3′, and reverse: 5′-ATCATTCTCTAGTGTCTGGTTGG-3′; IL-6 forward: 5′-AGGGCTCTTCGGGAAATGT-3′, and reverse: 5′-GAAGAAGGAATGCCCATTAACAAC-3′; IL-8 forward: 5′-ATGACTTCCAAGCTGGCCGTGGCT-3′, and reverse: 5′-TCTCAGCCCTCTTCAAAAACTTCTC-3′; TNF-α forward: 5′-CTCATCTACTCCCAGGTCCTCTTC-3′, and reverse: 5′-CGATGCGGCTGATGGTGTG-3′; IL-10 forward: 5′-GACTTTAAGGGTTACCTGGGTTG-3′, and reverse: 5′-TCACATGCGCCTTGATGTCTG-3′; β-actin forward: 5′-AGCGAGCATCCCCCAAAGTT-3′, and reverse: 5′-GGGCACGAAGGCTCATCATT-3′.

### Enyzme-linked immunosorbent assay

HPDLCs transfected with si-TRIM52 or si-Ctrl were exposed to 1 μg/ml LPS for 24 h. Afterward, the supernatants from HPDLCs were collected to evaluate the secretion of IL-6, IL-8, TNF-α and IL-10 using Human IL-6 ELISA kit, Human IL-8 ELISA kit, Human TNF-α ELISA kit, and Human IL-10 ELISA kit following the manufacturer’s instructions. The activity of caspase-3 was also determined by enyzme-linked immunosorbent assay (ELISA) kit (Abcam, Cambridge, U.K.) as recommended by the manufacturer.

### 3-(4,5-dimethylthiazol-2-yl)-2,5-diphenyltetrazolium bromide assay

HPDLCs were collected and cultured in a 96-well plate in the presence of 5% CO_2_ at 37°C. After 24 h of cultivation, HPDLCs were transfected with si-TRIM52 or si-NC followed by treatment with 1 μg/ml LPS for 0, 6, 12 and 24 h. Subsequently, HPDLCs were incubated with 3-(4,5-dimethylthiazol-2-yl)-2,5-diphenyltetrazolium bromide (MTT) solution (Sorlarbio, Beijing, China) for 2 h and then the absorbance was measured using a microplate reader at a wavelength of 490 nm.

### Flow cytometry

The apoptosis of HPDLCs was evaluated by flow cytometry using an Annexin V-FITC Apoptosis Detection Kit from Beyotime (Shanghai, China). After treatment, HPDLCs were collected, trypsinized and resuspended in Annexin V binding buffer. Therefore, HPDLCs were dual-stained with Annexin V-FITC and propidium iodide for 15 min in the dark at 4°C, and then analyzed by flow cytometry.

### Western blot

After treatment, HPDLCs were collected and lysed in RIPA buffer for protein extraction, as per the product’s instructions. Following sodium dodecyl sulfate/polyacrylamide gel electrophoretic separation, the extracted proteins were transferred on to polyvinylidene difluoride membranes. The membranes were blocked with 5% skim milk and then cultured with the primary antibodies against caspase-3 (Abcam), cleaved caspase-3 (Abcam), TLR4 (Abcam), p-TAK1 (Abcam), p-IKK-α/β (Abcam), p-IκBα (Abcam), NF-κB p65 (Abcam) and β-actin (Abcam) at 4°C overnight. Afterward, the membranes were immunoblotted with a secondary antibody conjugated to horseradish peroxidase (Abcam) for 1.5 h at room temperature. The immunoblots were detected by enhanced chemiluminescence from Pierce (Rockford, IL, U.S.A.) and quantified using ImageJ software (National Institutes of Health, Bethesda, MD, U.S.A.).

### Statistical analysis

Values were given as mean ± standard deviation. Statistical analysis was done using Student’s *t* test and one way-ANOVA from SPSS 22.0 software. Groups were deemed different when *P*<0.05 was obtained.

## Results

### Different concentrations of LPS induce the expression of TRIM52 in HPDLCs

**Figure 1:**
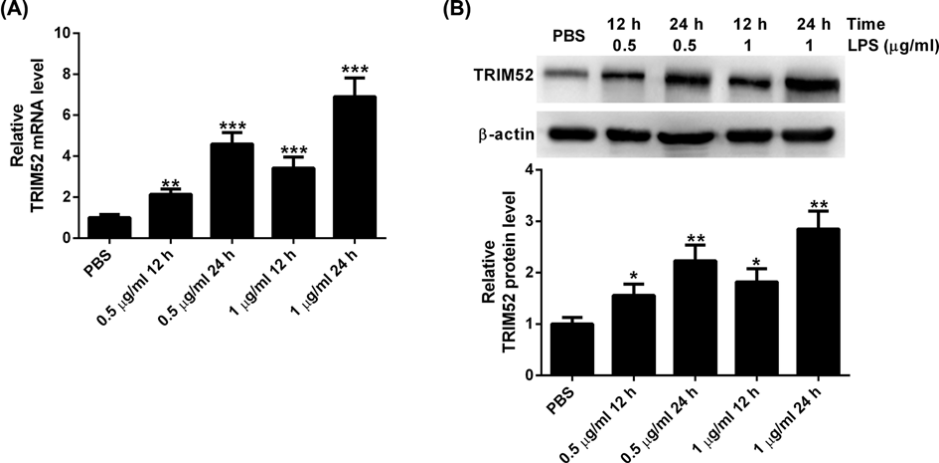
Different concentrations of LPS induce the expression of TRIM52 in HPDLCs HPDLCs were treated with different doses (0.5 and 1 μg/ml) of LPS. At 12 or 24 h after treatment, HPDLCs were tested for TRIM52 expression using qRT-PCR (**A**) and Western blot (**B**). *P<0.05, **P<0.01 and ***P<0.001.

To determine the role of TRIM52 in the LPS-induced inflammatory injury of HPDLCs in periodontitis, HPDLCs were treated with different doses of LPS, and then assayed for TRIM52 expression using quantitative real-time polymerase chain reaction (qRT-PCR) and Western blot. The results of qRT-PCR assay showed that the expression of TRIM52 was strikingly increased in HPDLCs treated with different doses of LPS (Figure 1A). In line with this, Western blot showed that different concentrations of LPS induced the up-regulation of TRIM52 in HPDLCs (Figure 1B).

### The silencing effect of siRNA on TRIM52

**Figure 2:**
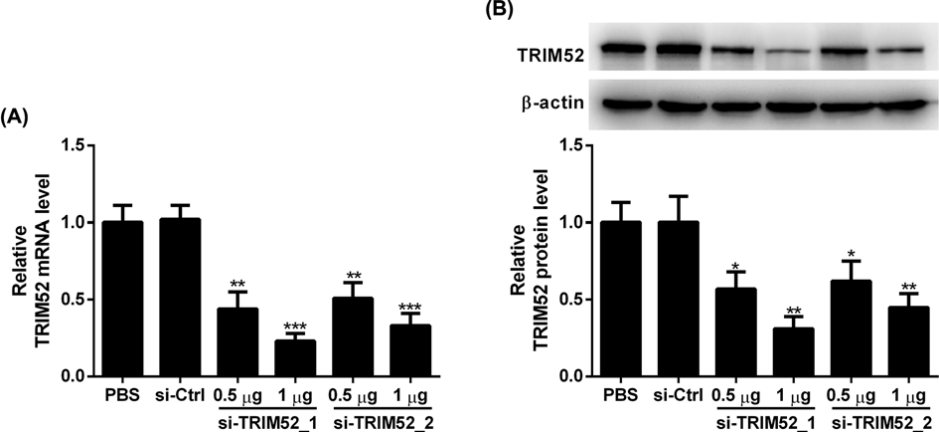
The silencing effect of siRNA on TRIM52 HPDLCs were transfected with si-TRIM52_1, si-TRIM52_2 or si-Ctrl, followed by culture for 24 h. (**A**) qRT-PCR and (**B**) Western blot were performed to detect the expression of TRIM52. *P<0.05, **P<0.01 and ***P<0.001.

Since TRIM52 was up-regulated following LPS treatment, we induced the down-regulation of TRIM52 by si-TRIM52 to investigate the role of TRIM52 in the LPS-induced inflammatory injury of HPDLCs. As determined by qRT-PCR, we found that compared with the si-Ctrl group, the expression of TRIM52 was remarkably reduced following si-TRIM52_1 and si-TRIM52_2 transfection in HPDLCs (Figure 2A). Similarly, transfection of si-TRIM52_1 and si-TRIM52_2 reduced the protein levels of TRIM52 in HPDLCs relative to the si-Ctrl group (Figure 2B). We chose si-TRIM52_1 with higher knockdown efficiency for subsequent experiments.

### Effect of TRIM52 knockdown on gene expression and secretion of IL-6, IL-8, TNF-α and IL-10 in LPS-induced HPDLCs

**Figure 3:**
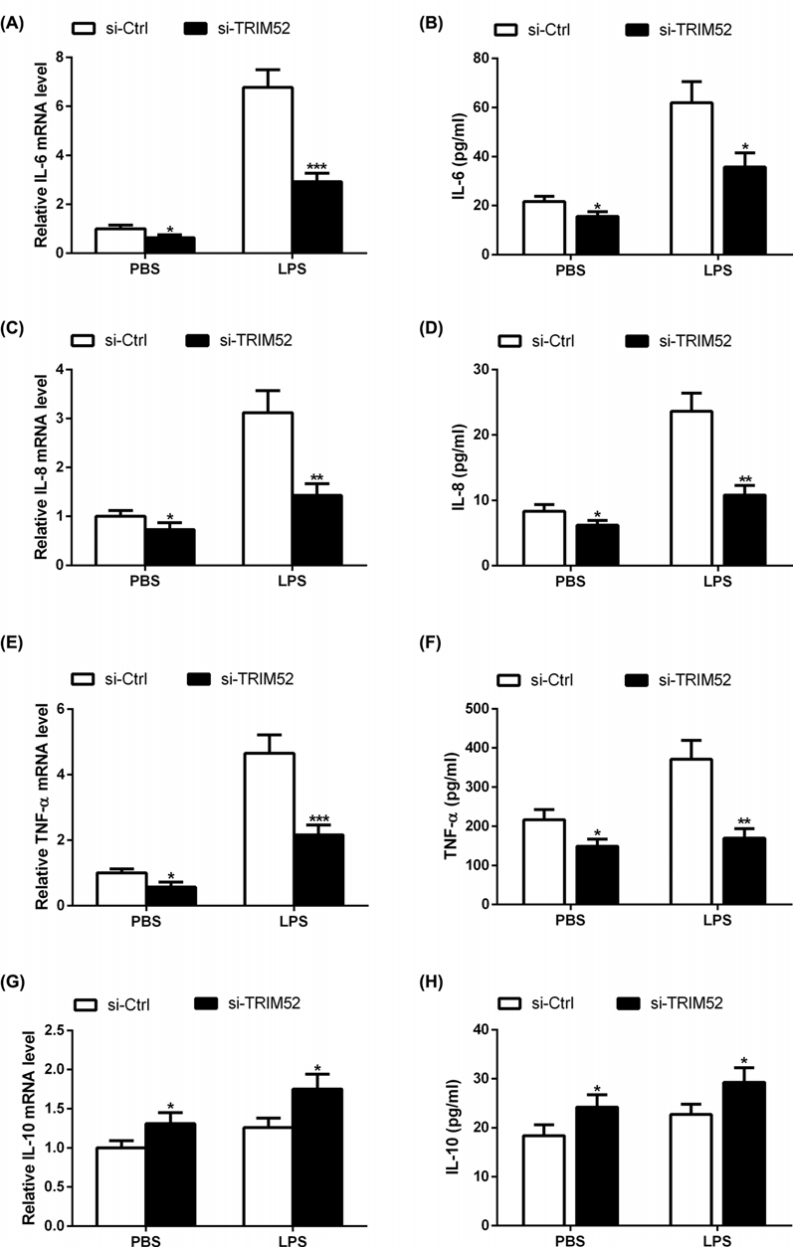
Effect of TRIM52 on gene expression and secretion of IL-6, IL-8, TNF-α and IL-10 in LPS-induced HPDLCs HPDLCs were transfected with si-TRIM52 or si-Ctrl, and then exposed to 1 μg/ml LPS for 24 h. The mRNA expression levels of IL-6 (**A**), IL-8 (**C**), TNF-α (**E**), and IL-10 (**G**) were determined using qRT-PCR. The secretion of IL-6 (**B**), IL-8 (**D**), TNF-α (**F**), and IL-10 (**H**) was detected using ELISA. *P<0.05, **P<0.01 and ***P<0.001.

To evaluate the effect of TRIM52 on the LPS-induced inflammatory response of HPDLCs, HPDLCs transfected with si-TRIM52 or si-Ctrl were exposed to 1 μg/ml LPS for 24 h, and the mRNA expression levels of inflammatory factors (IL-6, IL-8 and TNF-α), and anti-inflammatory factors IL-10 were examined using qRT-PCR. The results revealed that LPS administration increased the mRNA expression levels of IL-6, IL-8 and TNF-α in HPDLCs, which was attenuated following si-TRIM52 transfection (Figure 3A,C,E). In parallel, LPS stimulation induced the secretion of IL-6, IL-8 and TNF-α in HPDLCs, and these effects were blocked by silencing of TRIM52 (Figure 3B,D,F). TRIM52 knockdown promoted LPS-induced up-regulation of IL-10 mRNA and secretion of IL-10 in HPDLCs (Figure 3G,H).

### Effect of TRIM52 knockdown on LPS-induced proliferative inhibition and apoptosis in HPDLCs

**Figure 4:**
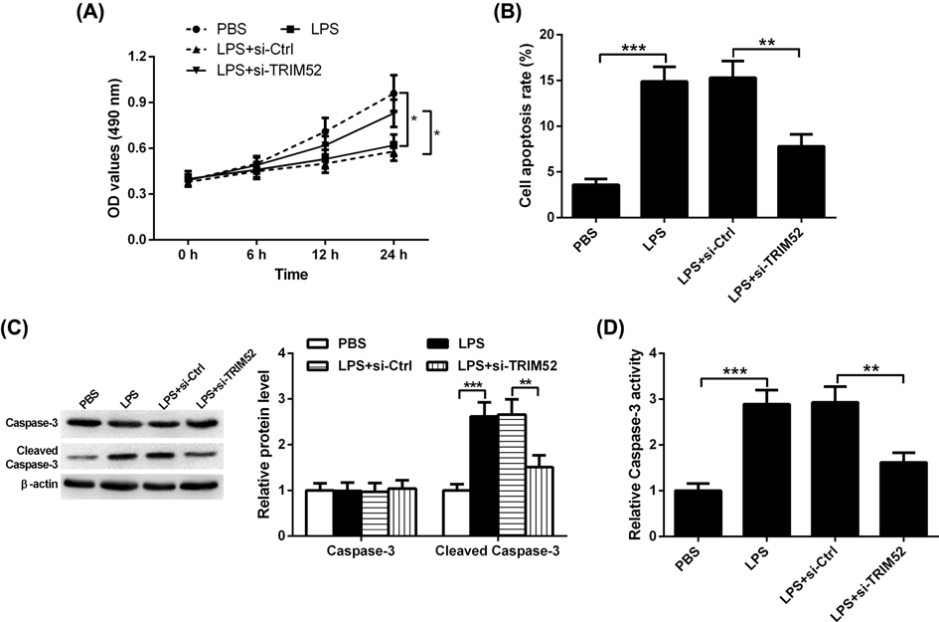
Effects of TRIM52 knockdown on LPS-induced proliferative inhibition and apoptosis in HPDLCs HPDLCs were transfected with si-TRIM52 or si-Ctrl, and then exposed to 1 μg/ml LPS for indicated times. (**A**) HPDLCs were tested for cell proliferation at 0, 6, 12 and 24 h after treatment using MTT assay. (**B**) The apoptosis of HPDLCs was assessed using flow cytometry. (**C**) The protein levels of caspase-3 and cleaved caspase-3 were evaluated using Western blot. (**D**) The activity of caspase-3 was evaluated using ELISA. *P<0.05, **P<0.01 and ***P<0.001.

The impact of TRIM52 knockdown on the proliferation of HDLCs in the presence of LPS was determined using MTT assay. As shown in Figure 4A, compared with the PBS group, the proliferation of HPDLCs was markedly suppressed following LPS treatment. Moreover, knockdown of TRIM52 abrogated LPS-induced proliferative inhibition in HPDLCs. Meanwhile, we found that LPS administration induced the apoptosis of HPDLCs, which was blocked by silencing of TRIM52 (Figure 4B). To further determine the mechanism by which TRIM52 silencing attenuates LPS-induced apoptosis in HPDLCs, the expression of caspase-3 and cleaved caspase-3 was detected by Western blot. HPDLCs stimulated with LPS exerted an increased level of cleaved caspase-3 protein. However, the up-regulation of cleaved caspase-3 triggered by LPS was abrogated after si-TRIM52 transfection. Notably, no change in caspase-3 protein level was discovered after LPS or LPS + si-TRIM52 treatment (Figure 4C). Besides, an obvious elevation of caspase-3 activity was noted in HPDLCs exposed to LPS, and this action was abolished by TRIM52 silencing (Figure 4D).

### Knockdown of TRIM52 represses TLR4/NF-κB pathway in LPS-treated HPDLCs

**Figure 5:**
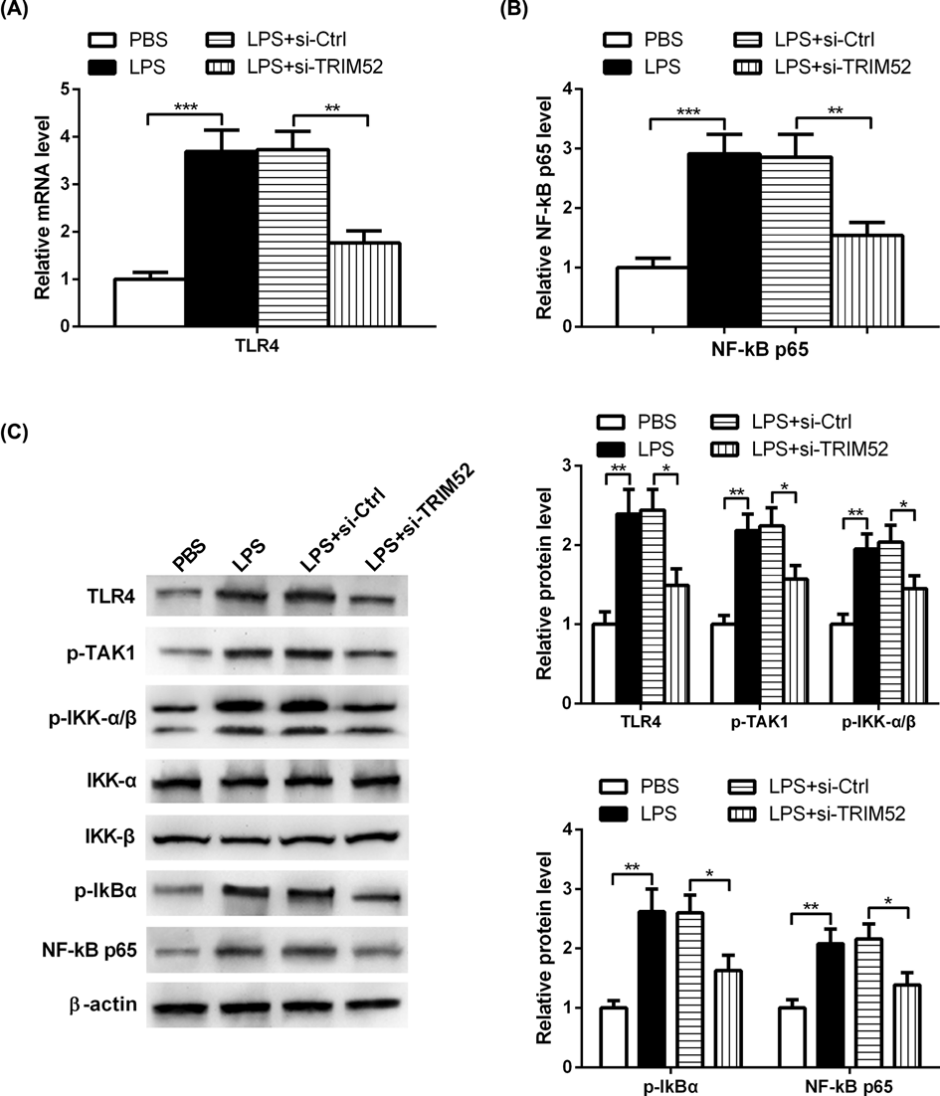
Knockdown of TRIM52 represses TLR4/NF-κB pathway in LPS-treated HPDLCs HPDLCs were transfected with si-TRIM52 or si-NC, and then exposed them to LPS or PBS. (**A,B**) qRT-PCR analysis showing that knockdown of TRIM52 attenuated LPS-induced elevation of TLR4 and NF-κB p65 expression in HPDLCs. (**C**) Western blot analysis showing that knockdown of TRIM52 attenuated LPS-induced elevation of TLR4, p-TAK1, p-IKK-α/β, p-IκBα and NF-κB p65 protein levels in HPDLCs. *P<0.05, **P<0.01 and ***P<0.001.

In order to further determine the signaling pathway involved in TRIM52-mediated progression of periodontitis, we transfected HPDLCs with si-TRIM52 or si-NC, and then exposed them to LPS or PBS. HPDLCs were collected and tested for TLR4 and NF-κB p65 expression using qRT-PCR assay. The results showed that the mRNA expression levels of TLR4 and NF-κB p65 were markedly increased in HPDLCs stimulated with LPS, and this increase was blocked when HPLDCs were treated with LPS and si-TRIM52 (Figure 5A,B). Consistently, the protein expression levels of TLR4, p-TAK1, p-IKK-α/β, p-IκBα and NF-κB p65 were strikingly increased in HPDLCs exposed to LPS, which was markedly attenuated after si-TRIM52 transfection (Figure 5C).

## Discussion

The pathogenetic mechanism of periodontitis is complicated and is far from clear. Periodontopantic bacteria-, especially LPS-, induced inflammatory injury has been documented to play a vital role in the development of periodontitis [17]. LPS can induce HPDLCs to secrete inflammatory factors, resulting in inflammation and tissue injury [18]. However, the genes involved in LPS-induced inflammatory injury have not been clarified comprehensively. Notably, TRIM52 has been demonstrated to be implicated in the host immune-inflammatory response. For instance, overexpression of TRIM52 promoted hepatitis B virus-induced fibrogenesis of LX-2 cells through phosphatase magnesium-dependent 1A-mediated Smad2/3 pathway [19]. Additionally, up-regulation of TRIM52 restrained the replication of Japanese encephalitis virus in BHK-21 and 293T cells by inducing the degradation of viral nonstructural protein 2A [20]. Although the antiviral activity of TRIM52 has been investigated in several studies, there is very little information on the impact of TRIM52 on LPS-induced inflammatory injury in HPDLCs. In our study, the up-regulation of TRIM52 was discovered in HPDLCs stimulated with LPS. Moreover, knockdown of TRIM52 alleviated LPS-induced proliferative inhibition and apoptosis promotion in HPDLCs, as evidenced by a decrease in cleaved caspase-3 expression and caspase-3 activity. Besides, silencing of TRIM52 suppressed LPS-induced inflammatory response of HPDLCs, as indicated by the reduction in IL-6, IL-8 and TNF-α. These findings suggested that TRIM52 acts a pro-inflammatory factor through enhancement of LPS-induced proliferative inhibition, apoptosis promotion and inflammatory response in HPDLCs.

TLRs are a class of transmembrane receptors, which have been recognized as a pioneer factor for the host immune response [21]. TLRs can identify and bind the pathogen-associated molecular patterns and then trigger the innate immune system to clear the invading pathogens [22]. As a member of TLR family, TLR4 has been identified as a LPS receptor and acts as a major player in LPS-induced inflammatory response. TLR4 is activated by LPS and then activates a cascade of signal pathways, leading to the production and release of inflammatory cytokines, such as IL-6, IL-8 and TNF-α [23]. For example, LPS-induced up-regulation of TLR4 promoted the expression of pro-inflammatory cytokines, and then suppressed the osteogenic differentiation and caused the adipogenesis of human periodontal ligament stem cells [24]. Furthermore, LPS stimulation time-dependently causes the generation of IL-6, IL-8 and TNF-α in stem cells from the apical papilla, which is blocked by TLR4 inhibitor, suggesting that TLR4 plays a vital role in LPS-induced inflammation [25]. Nevertheless, whether TRIM52 regulates LPS-induced inflammatory injury through targeting TLR4 has never been studied. Herein, we found that LPS treatment caused the up-regulation of TLR4 in HPDLCs. Moreover, TRIM52 knockdown could mitigate LPS-induced elevation of TLR4 expression, revealing that TLR4 is involved in the impact of TRIM52 on LPS-induced inflammatory injury during periodontitis.

TLR4, a transmembrane receptor, is responsible for the activation of a wide range of signal pathways involved in the bactericidal response [26]. Accumulating evidence has shown that the NF-κB signaling pathway serves as a crucial role in the progression of periodontitis [27]. Growing evidence indicated that TLR4-mediated NF-κB signaling plays an important role in the progression of periodontitis [28,29]. Previously, tormentic acid repressed LPS-induced generation of IL-6 and IL-8 through inhibiting TLR4-mediated NF-κB and P38 mitogen-activated protein kinase signaling [30]. Additionally, LPS treatment triggered the activation of TLR4-mediated NF-κB signaling, and then inhibited osteogenic differentiation of human periodontal ligament stem cells. Notably, blocking TLR4 or NF-κB signaling could partly overturn the impact of LPS on the osteogenesis potential of human periodontal ligament stem cells [31]. Similarly, LPS challenge increased the expression levels of TNF-α, TLR4, myeloid differentiation primary response 88, and NF-κB, and reduced the expression of IL-4 in HPDLCs, indicating that TLR4-mediated NF-κB signaling was implicated in LPS-induced inflammatory injury of HPLDCs [32]. Therefore, inhibition of TLR4-mediated NF-κB signaling is considered as an effective therapeutic approach for treating periodontitis. Remarkably, TRIM52 participates in regulating cellular function via the NF-κB pathway. Previously, down-regulation of TRIM52 repressed the proliferation and metastasis, and induced the apoptosis of SKOV3 and CAOV3 cells through regulating the NF-κB signaling [33]. However, if NF-κB signaling is involved in the regulation of TRIM52 in LPS-induced inflammatory injury is uncertain. In the present study, we found that the expression levels of p-TAK1, p-IKK-α/β, p-IκBα and NF-κB p65 were strikingly increased in HPDLCs exposed to LPS, while TRIM52 silencing restrained LPS-induced activation of NF-κB signaling, which is consistent with a prior study showing that TRIM52 functioned as a positive regulator of NF-κB signaling pathway [34].

Taken together, we found that TRIM52 was up-regulated in HPDLCs stimulated with LPS. Mechanically, knockdown of TRIM52 could mitigate LPS-induced inflammatory injury in periodontitis through TLR4/NF-κB signaling. These findings contributed to a better understanding of the inflammatory mechanism of periodontitis and indicated that TRIM52 may be a promising therapeutic target for periodontitis.

## Abbreviations

ELISA: enyzme-linked immunosorbent assay
HPDLC: human periodontal ligament cell
IL: interleukin
LPS: lipopolysaccharide
Mtb: *Mycobacterium tuberculosis*
MTT: 3-(4,5-dimethylthiazol-2-yl)-2,5-diphenyltetrazolium bromide
NF-κB: nuclear factor-κ B
p-IKK-α/β: phosphorylated IκB kinase α/β
p-IκBα: phosphorylated IκBα
p-TAK1: phosphorylated transforming growth factor-β-activated kinase
qPCR: quantitative real-time polymerase chain reaction
qRT-PCR: quantitative real-time polymerase chain reaction
siRNA: small interfering RNA
TNF-α: tumor necrosis factor-α
TRIM: tripartite motif-containing
